# The effects of flavonoid supplementation on the mental health of postpartum parents

**DOI:** 10.3389/fgwh.2024.1345353

**Published:** 2024-03-20

**Authors:** Rebecca Logan Colombage, Sean Holden, Daniel Joseph Lamport, Katie Louise Barfoot

**Affiliations:** School of Psychology and Clinical Language Sciences, University of Reading, Reading, United Kingdom

**Keywords:** mental health, postpartum, diet, flavonoids, polyphenols

## Abstract

**Introduction:**

During the postpartum period, parents face psychological challenges and consequently, changes in mood and associated mood disorders have become increasingly prevalent in the 6-months following birth. Dietary flavonoids have been found to benefit mood and are therefore an appealing non-pharmacological option for potentially treating mood disorders in the postpartum. The aim of this study was to investigate whether a two-week dietary flavonoid intervention would improve mothers’ and fathers’ mental health in the immediate 6-month postpartum period.

**Method:**

The study employed a randomised, parallel groups, controlled design to explore the effects of a flavonoid intervention vs. control group on several outcomes, including mood (PANAS), postpartum depression (EPDS), postpartum anxiety (PSAS-RSF-C) and quality of life (WHOQOL). Sixty participants (mothers *n* = 40, fathers *n* = 20) in the 6-month post-partum period were randomised to either a “flavonoid” or “control” condition. The flavonoid group were asked to add two flavonoid-rich foods (approximate flavonoid intake 218 mg/day) into their daily diet whilst controls (*n* = 23) were asked to continue with their usual diet for two-weeks (ClinicalTrials.gov (NCT04990622).

**Results:**

Significant effects were found in the flavonoid group where mothers reported higher positive affect and lower postpartum depression after the two-week intervention relative to baseline. This finding is especially relevant as a clinical reduction in postpartum depression scores in the flavonoid group by an average 2.6 scoring points was observed, which equated to a reduction from “possible depression” at baseline to “little or no depression” at 2-weeks, which was not observed in the control group. Fathers’ data was not analysed due to non-compliance with the intervention.

**Discussion:**

This study provides evidence for the benefits of a dietary flavonoid intervention for mood and mental health in new mothers, supporting the utility of non-pharmacological, self—administrable changes to the diet for improving positive mood outcomes and reducing symptoms of postpartum depression in mothers during an especially challenging time. Further research for the effect of dietary interventions on paternal mental health is needed.

**Clinical Trial Registration:**

ClinicalTrials.gov, identifier NCT04990622.

## 1 Introduction

Flavonoids, a bioactive plant compound found in fruits, vegetables and beverages, have been well cited for their beneficial effects on physical health (1–3) and cognition (4, 5). In addition, systematic reviews have highlighted the overall effects of flavonoid-rich foods on mood (6–8), whereby supplementing participants with flavonoid-rich foods, or extracts, can result in improvements to mood.

Mood benefits have been associated with a range of flavonoid-rich foods across the lifespan. Specifically, effects of flavonoid intervention have been observed acutely over a two-hour period following cocoa consumption (499 mg flavanols) (9) and chronically after 30-day cocoa supplementation in healthy middle-aged adults (500 mg polyphenols/day) (10). Furthermore, berry flavonoids have also been found to improve mood (specifically positive affect) following an acute study in 7–10 year old children (253 mg anthocyanins) (11). In addition to positive affect, flavonoid-rich foods have shown to hold benefits for longer, chronic psychological outcomes such as anxiety and depression. Fisk et al. (12) demonstrated a reduction of depression symptoms after a 4-week wild blueberry intervention (253 mg anthocyanins/day) in adolescents (aged 12–17). Similarly, this finding has been reflected in older adults. Esmaeilpour-Bandboni et al. (13) found a reduction of depressive symptoms in an older population after a five-week green tea intervention (dose not specified). In addition, Calapai et al. (14) showed a significant reduction in anxiety scores following a 12-week grape extract intervention in healthy older adults (250 mg capsule containing V. vinifera extract consisting of proanthocyanidins (>9% w/w) and anthocyanins (4%–5% w/w)/day). Collectively, these findings suggest that a variety of flavonoid-rich foods and extracts could benefit mood and mental health over the lifespan.

Evidence from studies exploring habitual diet also demonstrate protective effects of flavonoid-rich foods on mental health. Higher consumption of fruits and vegetables has shown to promote psychological well-being and mental health in longitudinal studies (15, 16). Furthermore, in a large systematic review, Głąbska et al. (17) found that a higher intake of fruits and vegetables, specifically flavonoid-rich berries, citrus fruits, and leafy green vegetables, reduced levels of psychological distress and had a protective effect against depression, supporting the idea that a greater consumption of flavonoid-rich foods in the habitual diet can promote mental health. Chang et al. (18), Godos et al. (19) and Park et al. (20) also found that high dietary intake of foods rich in proanthocyanidins, flavones, flavanones, and anthocyanins found in berries, citrus fruits and leafy green vegetables, were associated with a reduction of depressive symptoms in a dose response manner. These results indicate that both a higher intake and combination of foods rich in specific flavonoid subclasses could elicit optimal mood effects.

The majority of flavonoid and mood research has been conducted in healthy adults, though flavonoid interventions could be of particular benefit to populations who are susceptible to low mood such as new parents, who are at risk of Postpartum Depression (PPD). PPD is classified as an episode of Major Depressive Disorder [MDD; (21)], lasting longer than two weeks, with onset either during pregnancy (peri-partum onset) or within the first six months to one year following birth [post-partum onset; (21)]. However, the period during which PPD may occur is subject of discussion (22). While the DSM-5 confines the diagnosis of PPD to the initial four weeks after giving birth (21), others propose symptoms can manifest up to one year postpartum (23). While there is discrepancy regarding when PPD arises, there is consensus that PPD is often higher in the initial 6 months (24, 25) which then decreases over 18 months postpartum (26). Despite discrepancies of when PPD arises, research suggests up to 10%–15% of new mothers are affected by PPD, however this number is likely to be far greater as those experiencing symptoms don't often seek a diagnosis (27). Considering this, the period between birth and six-months represents a potentially sensitive period which may benefit from an intervention targeted at preventing mood disturbances.

As PPD is a type of MDD, symptoms are like those of other depressive disorders, including but not limited to feelings of irritability, lack of energy and feeling low or sad. As such, treatment for PPD is very similar to the treatment of depression, where antidepressants and psychological therapies are the main treatment routes. However, these options pose barriers for the postpartum population where there is varying accessibility, success rate and cost-effectiveness. Several risk factors are additionally associated with postpartum mood disorders; maternal age, where both older and younger mothers are higher risk (28, 29); prior history of depressive and anxiety disorders (30); having multiple children (31) and complications with birth, breastfeeding and pregnancy (32, 33), indicating higher risk individuals may benefit the most from treatment or prevention of mood disturbance within the postpartum.

PPD not only affects the mother, but also the family unit. Letourneau et al. (34) highlight that mothers with PPD may have reduced quality of maternal-infant interactions which have been shown to negatively impact infant development, in addition to affecting the relationship with their partner, highlighting the additional collective importance of treating and reducing the prevalence of postpartum mood disorders. Moreover, much of the PPD literature focuses on mothers, however nearly 1 in 10 new fathers (35) experience symptoms of PPD across the postpartum period. This indicates that paternal and maternal mental health should be equitable in prevention or treatment of PPD. In 50% of cases, maternal depression is accompanied by paternal depression (36), suggesting PPD can affect both caregivers of the family simultaneously. Poor paternal mental health is rapidly becoming a public health issue alongside maternal mental health due to its prevalence, impact on child development and on the healthy functioning of the family unit (37).

Prevention or treatment of paternal PPD is rarely considered; subsequently, research exploring flavonoid intervention in new parents should target both mothers and fathers to address the equivalent risks of low mood in both populations. Further, research suggests that lifestyle interventions focusing on families as a whole may be more feasible compared to existing approaches that primarily target individuals (38) likely due to shared motivation and social support (39). It is also significant to explore postnatal mood among fathers, or those in father roles in non-traditional family structures such as those with step-fathers and blended families, where instances of paternal depression have been observed to be more prevalent compared to traditional family structures (40). For the purposes of this study, the focus was postpartum mood in biological mothers and fathers, which remains as the most prevalent family structure of the UK population.

Consideration of mood beyond those diagnosed with postpartum mood disorders is also necessary as low mood, high anxiety and poorer mental health in non-clinical populations have been found to predict onset of postpartum mood disorders (41). Therefore, it may be beneficial to target interventions that focus on non-clinical populations to potentially prevent severity of symptoms that may contribute to the development of mood disorders during this sensitive period.

In view of the evidence for flavonoids and mood, flavonoid-rich foods may offer protection against onset or symptom severity of PPD. Recent evidence has shown that a dietary flavonoid intervention can promote mood regulation in a postpartum population. Barfoot et al. (42) conducted a randomised control trial where 0–12-month postpartum mothers were randomised to a flavonoid intervention group who added one additional flavonoid-rich food item to their normal diet every day for two weeks alongside a control group, who continued with their normal dietary routine. Mothers in the flavonoid group showed significantly reduced state anxiety and increased perceived quality of physical health highlighting that dietary flavonoid intervention has potential to improve mental health and wellbeing in the postpartum period.

The current study follows on from Barfoot et al. (42) by utilising the same two-week parallel groups design, and the same flavonoid-rich intervention foods to investigate postpartum mood. The current study focuses specifically on the 0–6-month postpartum period due to this being a particularly sensitive window for detecting PPD where mood is more labile. This will identify whether a dietary flavonoid intervention closer to birth, where onset risk is higher, offers greater benefits. Additionally, Barfoot et al. (42) required participants to consume one flavonoid item per day, whereas the present study increases this to two items per day. Previous research suggests a dose dependency of flavonoids, whereby a higher flavonoid dose translates to improved mood (18, 19, 43). Therefore, a higher dose may increase sensitivity to detect mood benefits, or benefits observed may be greater.

Furthermore, PPD-specific measures of depression and anxiety (Edinburgh Postnatal Depression Scale and the Postnatal-Specific Anxiety measure) are used in the current study which may increase sensitivity to change in postpartum mood compared to the Patient Health Questionnaire [PHQ-8; (44)] previously used by Barfoot et al. (42). Finally, the current study also recruits a small sample of fathers to pilot whether the dietary intervention would be feasible for fathers during this critical time. Therefore, the aim of this study was to investigate whether a two-week dietary flavonoid intervention would improve parents’ mental health in the postpartum period. Based on Barfoot et al. (42), we hypothesised that mothers and fathers in the flavonoid intervention arm would have a reduction in symptoms of state anxiety and improved quality of life. Due to the increased sensitivity of the other measures, we also hypothesised improved mood and reduced postnatal depression and anxiety as a result of the flavonoid intervention.

## 2 Methods

### 2.1 Design

The study employed a randomised, parallel groups, controlled design to explore the effects of a two-week flavonoid intervention vs. control group on several outcomes. The primary outcome measures for the study were state anxiety (State-Trait Anxiety Inventory-State scale; STAI-S) and depressive symptoms (Edinburgh Postnatal Depression Scale; EPDS). Secondary outcome measures included quality of life (World Health Organisation Quality Of Life; WHOQOL), postpartum specific anxiety (Postpartum Specific Anxiety Scale- Research Short Form; PSAS-RSF-C), current affect (Positive And Negative Affect Schedule; PANAS) and general diet (European Prospective Investigation of Cancer- Norfolk-Food Frequency Questionnaire; EPIC-FFQ). Outcome measures were assessed at two time points; Baseline (Day 0) and Post Intervention (Day 15). Using a random number generator, participants were randomly assigned to either a “flavonoid” group or a control group.

### 2.2 Participants

A priori-power analysis based on Barfoot et al. (42) rendered a total sample size of 40 participants to achieve a small effect (0.3) at a power of 0.95 and alpha level of 0.05. To pilot the feasibility of the intervention with fathers, a sample of twenty fathers in the 0–6 month postpartum were also recruited.

The primary inclusion criteria were that participants were a biological mother or father to an infant between 0 and 6 months old. Participants were excluded from the study if they had cancer, or conditions affecting the liver, heart or kidneys due to the unknown interactions of flavonoids with these conditions.

### 2.3 Mothers sample

**Figure 1:**
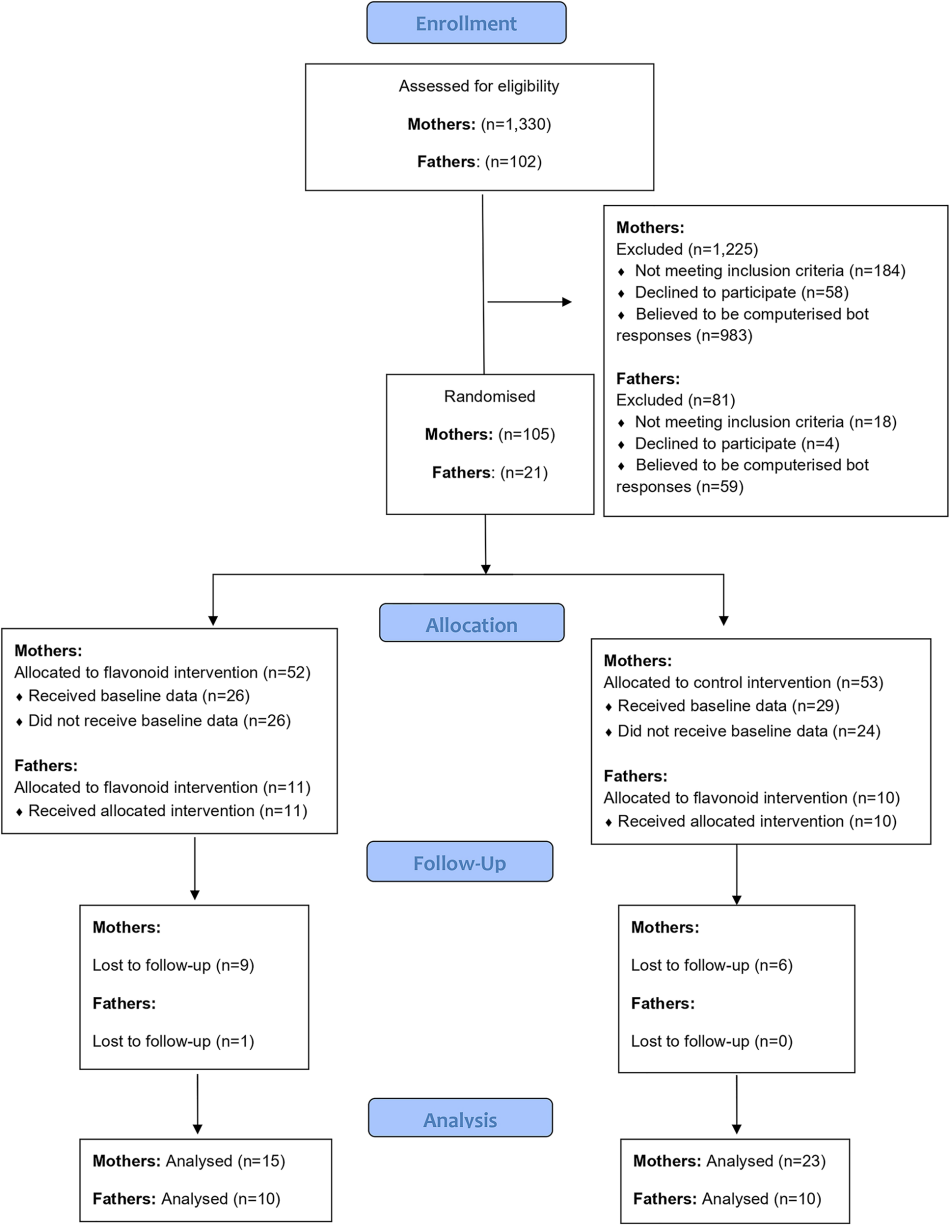
Consort diagram of participant recruitment.

One thousand, three hundred and thirty responses to the online advertisements were received via mum and baby pages on social media and in-person mum and baby groups in Berkshire during a seven-month period of recruitment. Of these, 1,125 were excluded (the majority due to being identified as computerized bot responses, see Figure 1). A total of 55 participants completed baseline data and 40 participants completed the intervention (See Figure 1). At data analysis, two participants (*n* = 2 flavonoid condition) were identified as outliers (outside interquartile ranges) for all mood outcomes both at baseline and post intervention, leaving *n* = 38 participants for analysis.

Of the sample of 38, 78.9% were White or Caucasian, 7.9% Asian or Asian British, 5.3% Black/African/Caribbean/Black British and 7.9% mixed race women, with 94.7% reporting to be married or in a domestic partnership (2.6% single and 2.6% divorced). Half the women had a bachelor's degree as their highest level of education (50%), 50% worked full time where 40% of the sample's occupation was medical professional, with 78.9% earning over £51,000. As per the study criteria, all women had a baby under six months old. For 17 (44.7%) women, this was their first child. In those who reported other children, the age of children ranged from 2 to 11 years. Twenty-two women (57.9%) reported nearby child support. Seven women (18.4%) reported a physical health diagnosis, with the most common being Eczema (5.3%). For psychological health, 4 participants (10.5%) reported a psychological diagnosis, of which, 1 woman had anxiety, 2 had postpartum depression and 1 had comorbid anxiety and depression. Out of these four women, two were taking medication for their mental health. In addition to this, 31 women (81.6%) took vitamins and supplements, which was majority Vitamin D (31.2%), multivitamin supplement (20.8%) and folic acid (18.2%) (See Table 1 for further demographic details).

**Table 1:** Demographic data for both mothers and fathers in flavonoid and control groups collected at baseline.

Measures	Mothers (*n* = 38)		Between groups *p*-value	Fathers (*n* = 20)		Between groups *p*-value
	Control group (*n* = 23) mean (SD)	Flavonoid group (*n* = 15) mean (SD)		Control (*n* = 10) mean (SD)	Flavonoid (*n* = 10) mean (SD)	
Age of parent (years)	35.82 (4.04)	34.40 (3.50)	.852	34.80 (4.29)	32.11 (3.79)	.168
Sleep of parent (hours per night)^a^	5.82 (1.32)	6.50 (0.52)	.098	7.17 (1.41)	7.15 (1.41)	.158
Age of baby (weeks)	15.82 (8.53)	14.60 (4.64)	.330	15.50 (9.66)	18.60 (6.15)	.421
Sex of baby (male: female)	18:5	8:7	.106	6:4	7:3	.639
Term of baby^b^	4:2:14:2:1	2:2:7:4:0	.538	1:2:5:2:0	2: 2: 3: 3:0	.793
Feeding method^c^	16:4:3	10:0:5	.111	4:0:6	5:0:5	.653
Specific diet^d^	0:0:1:1:0:21	1:1:0:1:112	.319	0:6:0:0:0:4	2:2:0:0:0:6	.273
Psychological diagnosis (yes: no)	2:21	2:13	.649	1:9	2:8	.531
Physical health condition (yes: no)	4:19	2:13	.737	0:10	1:9	.305
Other children (yes: no)	11:12	10:5	.254	4:6	5:5	.653

^a^ Assessed by asking parents to estimate, on average how many hours of sleep they estimate to get per night.

^b^ Very early term (born between 32 and 36 weeks): Early term (born between 37 and 38 weeks): Full term (born between 39 and 40 weeks): Later/Post term (born 41 weeks +): Other.

^c^ How the baby was fed for the first six-months (Breast milk: Formula: combination).

^d^ Parents following specific diets (Vegan: Vegetarian: Pescatarian: Dairy free: Gluten free: None).

To capture other risk factors, questions regarding pregnancy, birth and breastfeeding were included. These open-ended questions simply asked, “have you had any complications during your most recent birth experience?” meaning any medical complications or deviations from the birth plan, “have you had any complications during your most recent breastfeeding experience?” and “how do you feel towards your recent birth experience?”

### 2.4 Fathers sample

**Figure 2:**
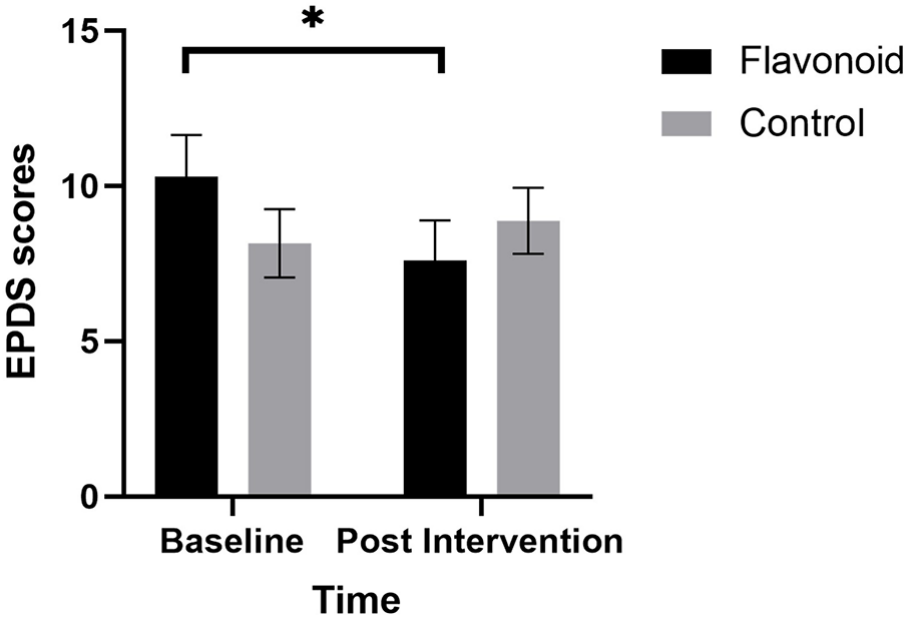
Linear mixed models with subjective sleep, maternal age, and baseline habitual flavonoid intake as covariates, revealed that mean (SEM) postpartum depression (EPDS) was significantly lower for mothers in the flavonoid group (*n* = 15) at 2-weeks compared to baseline (significant condition × time interaction F[1,37] = 10.38, *p* = .003). Bonferroni corrected post-hoc analysis indicated a significant reduction in depression in the intervention group (*p = .002) which was not reported in the control group (*n* = 23).

One hundred and two responses from interested participants were collected from parent pages on social media and word of mouth during a seven-month period. Of which, 81 bots were excluded, and 21 fathers completed baseline measures, with one drop out over the intervention (See Figure 2). Three participants from this group were partners of the mothers that also took part in the study. Nineteen participants were White or Caucasian (95%), with one participant categorising as Asian or Asian British (5%). All participants in the sample reported being married or in a domestic partnership. For education, 60% had a bachelor's degree and 50% were employed full time with 50% having a household income between £21,000 and £30,000 (see Table 1 for more demographic details).

All men recruited had an infant under six months, for 11 participants, this was their first child. For the remaining nine who reported having other children, age of children ranged from 4 to 15 years old. Childcare support outside the family home was reported to be greater compared to mothers, with 17 men (85%) reporting nearby childcare support. Three participants reported a psychological diagnosis, of which, all three reported depression. One participant reported a physical health condition (Asthma), and one participant reported taking medication, which was not described in more detail. Furthermore, 25% participants said they were taking a vitamin or supplement at time of the intervention which ranged from multivitamins, calcium and a protein supplement.

### 2.5 Intervention

Those in the flavonoid group were asked to continue with their current diet, but to add at least two high flavonoid food items per day (see Table 2) into their current diet. Comparatively, controls were asked to continue with their usual diet for two weeks. The items and portion sizes listed were taken from Barfoot et al. (42) which were chosen based on their flavonoid content, likability, accessibility, affordability, and typical portion size. The rationale for a variety of flavonoid-rich foods was to enable participants to choose from a range of foods which were likeable to the individual and easily available/affordable. Participants in both groups were asked to keep a food diary for the 2-week intervention, where the intervention group were asked to highlight the added flavonoid foods which enabled compliance to be tracked.

**Table 2:** Flavonoid items recommended to intervention group.

Flavonoid items	Estimated mean flavonoid mg/portion (45)	Number of occasions item was added over 2-weeks in the mothers group (*n* = 15)
		Mean (SD)
Berry fruits (∼120 g) e.g., blueberries, raspberries, strawberries, blackberries, blackcurrants, mixed berries	278	7.66 (5.08)
1 glass (250 ml) of fresh orange or grapefruit juice (not from concentrate)	54	6.30 (6.17)
2 large squares of dark chocolate (at least 70% cocoa)	47	6.06 (5.00)
4–5 cups of coffee (normal or decaf varieties) (250 ml)	1.215	4.77 (8.56)
1 portion of leafy green vegetables such as spinach or cabbage (∼70 g)	42	4.91 (2.34)
4–5 cups of tea (black or green) (250 ml)	894	2.11 (3.21)
1 large glass of red wine (250 ml)	206	1.92 (2.70)

In the mother sample, berry fruits were the most consumed item followed by fruit juice (See Table 2). One participant missed one day of the fourteen-day intervention, indicating good compliance (99.5%). However, the father sample did not complete adequate food diaries, whereby diaries were often too vague to understand what specific foods were consumed during the intervention, and therefore compliance was unclear for many fathers (55%). Therefore, the father's data was not of sufficient quality to undertake a reliable analysis.

### 2.6 Measures

#### 2.6.1 Demographics

At the Baseline timepoint (Day 0) (Table 1) data were collected on mother's age and age and sex of the baby. Sleep was self-reported by asking participants to estimate their subjective hours of sleep on average per night. Participants were asked what term the baby was born at, indicated by weeks of pregnancy as multiple-choice answers e.g., Full term, born between 39 and 40 weeks of pregnancy. Participants were asked how they had been feeding their baby using multiple choice answers of either “Breast milk”, “Formula milk”, “Combination feeding” or “Prefer not to say”. Specific diet was indicated by asking participants to note any dietary choices or restrictions e.g., Gluten free or Vegetarian. Participants stated whether they had a psychological or physical health diagnosis by selecting either “Yes”, “No” or “Prefer not to say” and giving the participant the option to disclose their physical or psychological health diagnosis. The same question was also asked in reference to whether they were taking medication for psychological or physical health reasons. Finally, we simply asked participants if they had any other children, where participants selected either “Yes” or “No” and had the option to add the number of children and children's age.

#### 2.6.2 Positive and negative affect schedule

To investigate mood, the Positive and Negative Affect Schedule [PANAS-NOW; (46, 47)] was used. This is a self-report measure consisting of twenty different words to describe feelings and emotions. Ten words reflect positive affect (PA), for example “interested” and “excited” and ten words reflect negative affect (NA), for example “guilty” and “scared”. The questionnaire is scored on 5-point Likert scale from 1- “Very slightly or not at all” to 5- “Extremely” in order to measure participants’ feelings at that present time. Scores are then summed to produce a score for each domain of positive and negative affect (scores range from 10 to 50) where higher scores indicate higher positive and negative affect. The PANAS is a reliable measure which is sensitive for flavonoid interventions (48) and is widely used in postpartum mood research.

#### 2.6.3 Edinburgh postnatal depression scale

The Edinburgh Postnatal Depression Scale [EPDS; (49)] is a 10-item self-report measure which can indicate symptoms of depression in a postpartum sample. Responses are on 4-point Likert scales relating to how they have been feeling in the last 7 days on a range from “Yes, most of the time” to “Not at all”. Results are scored from 0 to 30, with higher scores reflecting higher depressive symptomatology and a score of 10 being the clinical cut-off to indicate possible postpartum depression in women (49) and men (50). The EPDS is a reliable (51) and valid (52) measure and is one of the most widely used tools for measuring postpartum mood.

#### 2.6.4 Postnatal specific anxiety scale

To measure postpartum anxiety, the Postnatal Specific Anxiety Scale- Research Short Form for global Crises [PSAS-RSF-C; (53)] was used. This is a shorter version of the original, 51-item scale (54), which was validated for use during the COVID-19 crisis (2020–2022) which this study overlapped with (July 2021–Febuary 2022). Responses are scored on 5-point Likert scales, with possible responses including “Not at all; Sometimes; Often; Almost always; Not applicable”. After discounting any “Not applicable” scores, responses are summed with a maximum score of 48. Higher scores indicate higher anxiety, with 26 being a clinical cut-off to detect postpartum anxiety.

#### 2.6.5 World health organisation quality of life-BREF

In order to measure perceived quality of life, the World Health Organization Quality of Life-BREF [WHOQOL-BREF; (55)] questionnaire was used. The original WHOQOL-100 (56) was deemed too long for the present study. Therefore, the WHOQOL-BREF was included as an internationally used, validated and reliable measure of perceived quality of life. Furthermore, the WHOQOL-BREF has been recognized as a valid measure of quality of life in the postpartum period (57). The tool is a 26 item self-report measure of wellbeing over the last two weeks. Responses are scored on a 5-point Likert scale and are each summed into four domains; Physical health, Psychological, Social relationships and Environment. Three questions are negatively phrased and are subsequently reversed coded when scoring, raw scores are then transformed to represent standardized scores from the WHOQOL-100. Higher scores are indicative of higher perceptions of quality of life in each domain.

#### 2.6.6 State trait anxiety inventory

The STAI state scale [STAI-S; (58)] from the STAI was used. The STAI-S consists of 20 self-report questions to measure state anxiety. All items are rated on a 4-point Likert scale from “Not at all; Somewhat; Moderately so; Very much so”. Scores range from 28 to 80 with higher scores indicating higher state anxiety. A cut-off of 34 has been recognized to indicate state anxiety in postpartum populations (59).

#### 2.6.7 European prospective investigation of cancer food frequency questionnaire

In order to understand habitual nutrient intake, the European Prospective Investigation of Cancer (EPIC-Norfolk) FFQ (60) was used which is a valid and reliable for the measurement for habitual intake of food (61). The questionnaire measures average food consumption over the last year on a 9-point Likert scale from “Never or less than once per month” to “6 + times per day” for 127 food items. This measure was assessed at baseline and post-intervention; however, the wording was changed to reflect food consumption over a 2-week period rather than the last year. Nutrient intake was then calculated using the FETA software (62) from the EPIC FFQ, calculating participants’ average daily intake of various macronutrients, micronutrients and flavonoids (see Table 3).

**Table 3:** Mean (SD) raw data and interaction effects from the EPIC-norfolk FFQ for mothers in the flavonoid and control groups at baseline and 2-weeks post intervention.

Nutrient	RDA	One sample *t*-test comparing baseline intake with RDA'S	Baseline		Between groups *p* value	Post-intervention		Condition** × **time interaction
			Flavonoid (*n* = 15)	Control (*n* = 23)		Flavonoid (*n* = 15)	Control (*n* = 23)	
			M (SE)	M (SE)		M (SE)	M (SE)	
Flavonoid (mg)^a^	428 mg	t(35) = 2.25, *p < *.030	494.08 (144.79)	770.86 (119.44)	.149	1,606.860 (143.61)	1,519.12 (115.98)	F(1, 38)* = *2.42, *p = *.128
Calories (kcal)^b^	2,500 kcal	t(35) = −9.43, *p < *.001	1,618.31 (217.95)	1,756.08 (176.01)	.626	1,606.85 (143.61)	1,519.12 (115.98)	F(1, 38) = .55, *p = *.461
Protein (g)^b^	210 g	t(35) = −38.45, *p < *.001	60.41 (10.13)	76.34 (8.35)	.233	64.92 (6.60)	61.82 (5.33)	F(1, 38) = 1.95, *p = *.170
Fat (g)^b^	90 g	t(35) = −5.62, *p < *.001	64.71 (8.48)	72.76 (6.99)	.469	65.28 (7.44)	60.45 (6.01)	F(1, 38) = 1.01, *p = *.320
Carbohydrates (g)^b^	300 g	t(35) = −7.72, *p < *.001	208.24 (26.89)	232.18 (22.17)	.496	199.43 (17.30)	191.57 (13.97)	F(1, 38) = .794, *p = *.379
Fruit (g)^b^	400 g	t(35) = −7.78, *p < *.001	200.01 (125.92)	351.31 (103.96)	.360	198.27 (39.04)	218.62 (31.52)	F(1, 38) = .613, *p = *.439
Vegetables (g)^b^	400 g	t(35) = −7.20, *p < *.001	261.55 (229.59)	489.31 (189.55)	.449	396.44 (113.86)	204.72 (91.95)	F(1, 38)* = *1.71, *p = *.199

^a^ Vogiatzoglou et al. (63).

^b^ U.S. Department of Agriculture (64).

#### 2.6.8 Food diaries

All participants were sent a 14-day food diary template after completing baseline measures (PANAS-NOW, EPDS, PSAS-RSF-C, WHOQOL-BREF, STAI-S and EPIC FFQ). This instructed participants to list what they ate every day, over the intervention period. Participants allocated to the flavonoid group were asked to also note the additional intervention foods they included in their diet. To retain compliance with the intervention schedule, participants were not asked to weigh foods when recording food items in food diaries. In addition, the diaries were used to maintain engagement with the study as the study population needed easy procedures during this period of energy and time constraint. Including the food diary in both groups was an improvement from Barfoot et al. (42) such that both conditions had a matched design. A detailed analysis of the food diaries was not undertaken as this was outside the scope of the study, instead assessment of general diet was monitored with the FFQ. The food diaries were used to assess the nature and quantity of the flavonoids added to the diet in the intervention group and to encourage and monitor compliance.

### 2.7 Procedure

Participants signed up to the study via an online link. Eligible participants were randomised to the intervention or control group and sent links to the relevant first survey to record baseline data. Data collection was conducted on two separate occasions, baseline (day 0) and 2-weeks later (day 15).

At baseline, participants completed a demographics questionnaire (see Table 1), then the PANAS-NOW, EPDS, PSAS-RSF-C, WHOQOL-BREF, STAI-S and EPIC FFQ in a fixed order. When baseline data was complete, participants were presented with instructions for the intervention. Those in the intervention condition were instructed to add two additional foods (Table 2) into their diet per day, whilst controls were encouraged to not make any changes to their current diet, though all participants were instructed to log all foods consumed in a daily food diary. If participants had missed a day, they were encouraged to record it as “missed” and continue as normal the next day. Instructions and the food diary template were emailed to the participant after baseline measures were complete. Half-way through the intervention (day 7), participants were contacted by the researcher to check-in with the progress of the intervention. Finally, at day 15, participants were sent the second survey link which included all outcome measures (the PANAS-NOW, EPDS, PSAS-RSF-C, WHOQOL-BREF, STAI-S and EPIC FFQ), and participants were asked to email their food diaries to the researcher.

Email reminders were sent on day 16 and day 18 for any participants that had not completed the second survey (*n* = 6) or sent their food diaries. Upon debriefing, all participants were provided with helplines and weblinks specific to parental mental health support and were encouraged to contact their GP should they wish to seek further support. All participants were reimbursed with a £15 Amazon voucher. Data was collected between July 2021 and February 2022 and the study was given a favourable ethical opinion for conduct by the University of Reading School of Clinical Language Sciences Ethics Committee (2021-171-KB) and is registered at ClinicalTrials.gov (NCT04990622).

### 2.8 Data analysis

For the qualitative demographic questions, quantitative content analysis was undertaken for participants pregnancy, birth, and breastfeeding experiences whereby the number of yes responses were counted for those experiencing difficulties. For the question “How do you feel towards your recent birth experience”, data was categorised into whether participants had a more negative or positive birth experience overall, based on their qualitative descriptions.

Quantitative data was analysed using SPSS statistics (version 27). Independent groups *t*-tests and Chi-squared analysis were conducted to investigate whether there were significant group differences in demographic variables at baseline including parent age, subjective hours of sleep, sex of baby, term baby was born in (e.g., 39–40 weeks), method of feeding baby, specific diets consumed (e.g., vegetarian or vegan), whether the participants had diagnosed psychological or physical health problems, and whether the parents had other children. For PANAS-NOW (PA, NA), STAI-S, EPDS, PSAS-RSF-C, WHOQOL-BREF (physical, psychological, social, environmental), data were analysed using separate linear mixed models where Condition (flavonoid, control) and Time (baseline, 2-weeks) were fixed effects. An unstructured covariance matrix was used to model the repeat effects of Time with participants as random factors. Bonferroni-adjusted pairwise comparisons were used to explore significant interactions between and within groups, with the addition of covariates (subjective sleep, maternal age, and baseline habitual flavonoid intake). These covariates were chosen due to their relationship with mood which could potentially influence results. Subjective sleep duration was included as a shorter sleep duration and increased sleep disturbances have been well cited with lower levels of mood and wellbeing alongside a greater risk of developing mood disorders (65, 66). In particular, postpartum women often experience altered sleep patterns which may affect mood (67). Secondly, as discussed, maternal age is a significant predictor of postpartum mood, with older and younger mothers at a larger risk of poorer mood outcomes, hence age is an important factor to control for in the model. Finally, baseline habitual flavonoid intake was included as a covariate to account for the potential impact of habitual diet which may affect mood outcomes according to epidemiological data (20). Where covariates were significant predictors, further exploration was conducted using correlations between the covariate at Baseline, Post intervention and change from baseline (week 2—baseline) scores.

The same structured model was used to analyse the EPIC-Norfolk FFQ (flavonoids, calories, carbohydrate, protein, fat, fruit, vegetables) to explore whether enrolment in the dietary study led to any significant changes in habitual diet, macronutrient, and flavonoid intake. One sample *t*-tests were also conducted to explore differences in mean nutrient intake at baseline with the UK recommended dietary allowance (RDA) for mothers in the postpartum period to explore general diet quality in the sample and investigate whether mothers were meeting the RDAs at baseline.

## 3 Results

There were no significant differences in demographics (Table 1) and outcome data (Table 4) between flavonoid or control groups at baseline.

**Table 4:** Mean (SD) raw outcome variable data and interaction effects for mothers in the flavonoid and control groups at baseline and 2-weeks post intervention.

Mothers (*n *= 38)					
Measures	Baseline			Post intervention	
	Control (*n* = 23) (M, SE)	Flavonoid (*n* = 15) (M, SE)	Between groups *p*-value for baseline comparison	Control (*n* = 23) (M, SE)	Flavonoid (*n* = 15) (M, SE)
EPDS	8.16 (1.10)	10.29 (1.34)	.237	8.88 (1.05)	7.69 (1.29)
STAI-S	35.63 (2.04)	41.27 (2.11)	.095	34.90 (2.10)	36.39 (2.57)
PSAS-REF-C	22.04 (1.01)	21.93 (1.23)	.942	20.50 (1.00)	18.46 (1.22)
Positive affect	30.70 (1.76)	27.56 (2.15)	.277	30.66 (1.63)	31.96 (2.00)
Negative affect	16.71 (1.10)	15.82 (1.34)	.617	14.40 (0.95)	14.82 (1.16)
WHOQOL physical	63.08 (3.40)	63.07 (4.13)	.998	65.95 (2.63)	75.00 (3.20)
WHOQOL psychological	52.64 (4.11)	59.99 (4.99)	.266	47.05 (2.45)	50.65 (2.98)
WHOQOL social	50.57 (4.27)	55.41 (5.19)	.482	59.71 (3.56)	57.86 (4.35)
WHOQOL environmental	73.04 (3.24)	79.39 (3.96)	.231	72.14 (4.28)	67.59 (5.20)

A significant Condition × Time interaction (F[1,37]_ _= 10.38, *p = *.003), revealed that mothers in the flavonoid condition reported significantly lower postpartum depression at the end of the two week intervention compared to baseline (*p = .*002), which was not evident in the control group (*p = .*276). There were no significant main effects of Condition or Time or any covariate in the model (*p *> 0.05) (Figure 2).

**Figure 3:**
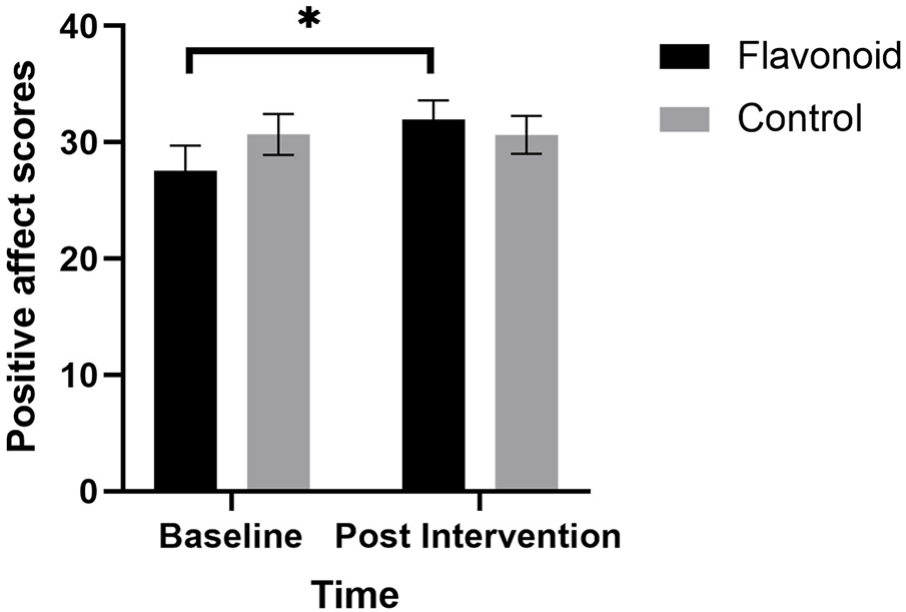
Linear mixed models with subjective sleep, maternal age, and baseline habitual flavonoid intake as covariates, revealed that mean (SEM) positive affect (PANAS) was significantly higher for mothers in the flavonoid group (*n* = 15) at 2-weeks compared to baseline which was not seen in the control (*n* = 23) with a significant main effect of Time F[1,37] = 4.92, *p* = .033 and significant condition × time interaction F[1,37] = 5.13, *p* = .029). Bonferroni corrected posthoc analysis indicated a significant reduction in depression in the intervention group (*p = .006) which was not reported in the control group (*n* = 23).

A significant Condition × Time interaction (F[1,37]_ _= 5.13, *p *= .029) was also found for positive affect where mothers in the flavonoid group had significantly higher positive affect scores at Week-2 compared to baseline (*p *= .006) which was not evident in the control group (*p *= .971) (Figure 3). Additionally, results revealed a significant main effect of Time, where positive affect was greater at follow up regardless of condition (F[1,37]_ _= 4.92, *p *= .033). Interestingly, Mothers age was a significant covariate (F[1,37]_ _= 4.62, *p *= .038). Age and positive affect scores were not significantly associated at baseline (*r* = −.098, *p *= .557), or change from baseline (*r* = .285, *p *= .083), though were marginally significant post-intervention (*r* = −.308, *p *= .060), suggesting older ages were associated with lower positive affect at the end of the study, regardless of intervention type.

State anxiety (F[1,37]_ _= 4.10, *p *= .05), negative affect (F[1,37]_ _= 4.78, *p = *.035), postpartum anxiety (F[1,37]_ _= 12.46, *p = *.001) and physical health quality of life (F([1,37]_ _= 5.07, *p = *.029) were significantly lower at follow up compared to baseline (a significant main effect of Time), however, interactions and main effect of Condition were not significant (*p > *0.05). For psychological, social and environmental quality of life scores no significant interaction or main effects were found (*p* > .05).

Regarding habitual diet, analysis of data from EPIC-Norfolk showed no significant differences between flavonoid and control conditions at baseline (Table 3). Both control (*p = *<.001) and flavonoid (*p = *<.001) conditions had a higher flavonoid intake post intervention compared to baseline (F[1,37]_ _= 63.09, *p = *<.001, see Table 3). Additionally, one sample *t*-tests were conducted to explore differences between mean nutrient intake at baseline and the UK recommended dietary allowance (RDA) for mothers in the postpartum period. Significant differences were observed for all nutrients (Table 3), whereby mothers were under-achieving RDA's for most macronutrient groups, except for flavonoid intake in both conditions, and vegetable intake in the control condition, where they were higher than the respective RDA (Table 3).

For the qualitative data, a total of 25 mothers provided responses regarding their health experiences during pregnancy. Among these, 18 (72%) mothers acknowledged facing pregnancy challenges. In relation to birth complications, 20 mothers shared their experiences, with all of them highlighting difficulties during childbirth (100%). Elaboration of the birth experience was given by all 38 participants, where 21 (55%) participants described their experiences as more positive, and 17 (44%) more negative. For breastfeeding, 27 mothers responded, all of which indicated encountering difficulties (100%).

## 4 Discussion

The current study recruited both mothers and fathers with infants under six months old with the aim to investigate whether a two-week dietary flavonoid intervention would improve parents’ mental health in the 6-month postpartum period. Mothers who consumed two additional flavonoid-rich foods per day over two weeks had a significant reduction in postpartum depression scores and a significant increase in positive affect post intervention relative to baseline. No such differences occurred in the control group. These results suggest that regular consumption of flavonoid-rich foods in the habitual diet during the first 6-months postpartum may alleviate symptoms of postpartum depression and improve transient mood in the postpartum population. Due to lack of compliance with the intervention and outcome measures, fathers’ data were not analysed, suggesting potential barriers to engagement in this sample. Overall, findings provide evidence to further support the idea that flavonoid-rich foods are an accessible and cost-effective option to promote mental health in mothers in the immediate postpartum period, where risk of mood disturbance is higher than that of the general population.

The overall improvement in positive affect for the flavonoid group aligns with findings from Khalid et al. (11) who found higher positive affect following acute blueberry intervention across separate samples of children and young adults. Considering that low positive affect is linked to depression and high negative affect is linked with anxiety (46, 47) it could be argued that acute and chronic affective states associated with depressive disorders are more sensitive to flavonoid interventions compared with those associated with anxiety, as both negative affect and anxiety measures did not significantly change as a result of the flavonoid intervention. Furthermore, sensitivity of a flavonoid effect on chronic depressive symptomology may also depend on the specificity of the measure used. Barfoot et al. (42) found no flavonoid-related outcomes on the PHQ-8, a measure of depression designed for the general population. However, the current study found significantly reduced depression following 2 weeks of dietary flavonoids using the EPDS, a tool specifically designed to capture PPD in a postpartum population. This highlights that though depressive states may be more sensitive to flavonoid interventions, the outcome measure used is also important, though additional research is needed in this population to further investigate effects of flavonoid supplementation on a range of mood states and underlying mechanisms which may drive this effect.

Maternal age was highlighted as a predictor of positive affect scores, whereby being an older mother was associated with lower positive affect scores post-intervention. In the study, 5 mothers were approaching 40 (aged 38–39) and 2 were over 40, suggesting an older subsample in the study that may have been associated with poorer mood, as has been observed in previous research (28). Upon further exploration, age was not significantly related to positive affect at baseline or change from baseline analysis. Considering these findings, it may be of interest to further explore whether the benefits of nutritional interventions for mood during the postpartum period are influenced by age.

At baseline, postnatal depression and postpartum anxiety (PPA) scores (as assessed by EPDS and PSAS respectively) were noticeably higher than the number of recorded mental health diagnoses in the sample (postnatal depression *n* = 1, postnatal anxiety *n* = 1, postnatal anxiety and depression *n* = 1 anxiety *n* = 1) whereby 42% of mothers scored over the clinical cut-off for PPD and 60% were above cut-off for PPA. This exceeds respective global population estimates [PPD: 10%–15% (27); postpartum anxiety: 13%–40% (68)], indicating symptoms indicative of PPD and postpartum anxiety are noticeably higher than the number of recorded mental health diagnoses in the sample. This highlights the importance of investigating postpartum mental health within the wider context where risk of missed diagnoses may be high. Furthermore, the qualitative data indicated that the percentage of women reporting a negative birth experience (44%) was also higher than previously recorded prevalence, ranging from 7 to 21% [(69); Henriksen et al., (70)]. It is unclear why this might be and future research should investigate the factors that might affect mothers’ interpretation of birth experience and relation to mood. These data may highlight that the sample were more clinically vulnerable than the general postpartum population. However, the significant reduction in mean postpartum depression scores in the flavonoid group from “possible depression” at baseline to “little or no depression” at 2-weeks (−2.6 scoring points) highlights the potential for a flavonoid-rich diet to alleviate some symptoms of clinical depression in this cohort.

In addition to benefits for postpartum depression and positive affect, results showed significantly lower state anxiety scores for both groups over the two weeks. In comparison, Barfoot et al. (42) found significant state anxiety effects for the flavonoid group only in postpartum mothers. The reason for differences in the STAI scores from Barfoot et al. (42) and the current study remains unclear, although highlights the need for further investigation into the effects of flavonoids in a postpartum sample. Despite this, both the STAI and PSAS-RSF-C seem to be somewhat sensitive to changes across time in this period which supports use of both measures in future postpartum populations.

The EPIC FFQ was utilised to assess baseline habitual diet and to monitor stability of participants’ diets over the 2-week intervention period. The only outcome from the EPIC FFQ to significantly change over the two-week intervention was flavonoid content, where flavonoid intake significantly increased from baseline to post-intervention in both groups. Flavonoids were not explicitly mentioned in study advertisements, rather diet was highlighted as the variable of interest, so this effect cannot be explained by a flavonoid knowledge bias. This finding may have been an effect of food diary completion leading to increased awareness of diet over the intervention period whereby participants consumed food items that happen to be rich in flavonoids. Reassuringly, the mean change between pre and post was highest for the flavonoid group compared to the control (Table 3). As no significant differences were found in other EPIC items such as fruit and vegetable intake it's likely participants consumed other flavonoid-rich foods and beverages such as cocoa and tea which contributed to the significant change in flavonoid consumption in both groups. However, these specific items were not captured in the EPIC analysis, making it difficult to ascertain the main sources of flavonoids in the participant's diet. It is also important to emphasise here that the food diaries did not provide enough data to accurately estimate flavonoid or other nutrient intake, and the main purpose, as previously described, was to identify the intervention food items, to assess and encourage compliance, and to match procedures across the two groups. Future research should consider use or development of other dietary measures which may provide better capture of high flavonoid foods, for example 24-hr food recalls (71) or a more comprehensive collection of flavonoid-rich items in current dietary measures.

Food diaries in the current study did accurately capture the two daily flavonoid-rich items participants added to their diet over the 2-week intervention period. Berry fruits, orange juice and dark chocolate were the most consumed intervention food by mothers (22%, 18% and 17% consumed over the two-week period respectively). Our results are in keeping with previous experimental and epidemiological studies demonstrating antidepressant effects of a high flavonoid, berry-rich diet (11, 12, 18). Regarding potential mechanisms, berry fruits are rich in anthocyanins, which have been hypothesised to have regulatory abilities for mood by crossing the blood brain barrier (BBB) acting as a monoamine oxidase (MAO) inhibitor (72, 73). Elevated MAO has been observed in the immediate postpartum period (4–6 days after birth) (74) which was later associated with postpartum depressive symptoms (75), suggesting the MAO hypothesis for depression may extend to the postpartum. Promising findings from Dowlati et al. (76) additionally showed increased resilience in depressed mood following a blueberry flavonoid dietary supplement in the 0–5-day postpartum period. Collectively, this evidence could explain why depressive mood and positive affect were improved in the present study following an increased consumption of berry fruits, though more research is warranted.

There is also evidence that flavonoids may regulate mood via maintaining neuroplasticity. It has been established that those with mood disorders, such as anxiety and depression have a reduction in circulating brain derived neurotropic factor (BDNF) [see (77, 78) for reviews]. A recent review from German-Ponciano et al. (79) concluded that flavonoid supplementation (minimum duration 14-days) in rodents has the ability to reverse depressive behaviours via increasing BDNF levels, highlighting flavonoid supplementation as a potential for the treatment in depression. Collectively, these results indicate that flavonoids may act as therapeutic candidates for mood disorders by maintaining neuroplasticity, potentially acting as the underlying mechanism in the current study. However, more conclusive research needs to be conducted with flavonoid supplementation, assessing BDNF levels and mood outcomes in humans with interventions lasting minimum 14-days to explore this.

As discussed, fathers’ data was not analysed due to lack of engagement with the study materials and intervention, meaning we were not able to conclude whether a flavonoid intervention benefited mood in fathers. This continues to be an area of research interest due to the significant risk of poor mental health and PPD in the immediate postpartum for fathers and male caregivers. Additionally, there was a large attrition rate (Figure 2), where fathers seemed less motivated than mothers to participate in a nutrition and mood study. Further research should explore engagement of fathers in nutritional interventions and paternal mental health, to evaluate whether certain study methodologies (e.g., online testing) or other factors around motivation for involvement or completion of the research affect engagement. Mitchell et al. (80) stress the difficulty in recruiting and retaining fathers in research, highlighting that high attrition rates in paternal research is common. Under engagement in research has also been associated with lower socio-economic status (81). Our father's sample had a slightly lower household income than UK average at £32,300 (82) and compared to our mothers sample, which may partly explain the lack of engagement in the trial. Additionally, majority of fathers (*n* = 18) did not have a partner also taking part in the trial which may have resulted in a lack of shared motivation and social support which perhaps could be a barrier for fathers engaging in nutritional interventions. Another major barrier to paternal research is recruitment, which is primarily conducted via their partners (83). Recent research suggests that studies seeking to recruit fathers for parenting research should prioritize using advertisements specifically tailored to fathers, claiming that 79% of participants recruited through father-targeted advertisements were male, while only 14% of participants recruited through advertisements targeting parents in general were male (84). Collectively, this highlights a growing need to explore paternal involvement with research generally and applying these methods to nutritional research. In addition, the structure of families within the UK is as varied as it has ever been, with only 44% of families in a traditional nuclear structure of a biological mother, father and child(ren) cohabiting (85), therefore is important to acknowledge that prevalences in parental mental health may vary depending on changing family structures. Furthermore, same sex couples have been found to have higher rates of postnatal depression than heterosexual couples (86) with research suggesting perinatal mental health may be poorer in Lesbian, Gay, Bisexual, Transgender, Queer or Questioning, Two spirit and additional sexual orientations and gender identity (LGBS+) couples (87). Exploring the mental health of parents and caregivers inside and outside the heteronormative realm is critical to supporting and treating PPD at population level.

In conclusion, this study provides evidence for the benefits of a dietary intervention for mood and mental health to mothers in the immediate postpartum. Specifically, this study demonstrated improvements in postpartum depression symptoms and positive affect following a 2-week flavonoid-rich diet in the 0–6-month postpartum. The research also observed that fathers in this critical period lacked adequate engagement, highlighting a need for further research into diet and paternal mental health.

## Data Availability

The raw data supporting the conclusions of this article will be made available by the authors, without undue reservation.
